# Assessing the Implementation of the Baby-Friendly Hospital Initiative in Hungary: A Cross-Sectional Study

**DOI:** 10.3390/children11040412

**Published:** 2024-03-29

**Authors:** Anita Hulman, Katalin Varga, Tímea Csákvári, Annamária Pakai

**Affiliations:** 1 Doctoral School of Health Sciences, Faculty of Health Sciences, University of Pécs, 4 Vörösmarty Str., H-7621 Pécs, Hungary; 2 Department of Affective Psychology, Institute of Psychology, ELTE Eötvös Loránd University, 46 Izabella Str., H-1064 Budapest, Hungary; 3 Department of Health Economics and Health Care Management, Institute of Health Insurance, Faculty of Health Sciences, University of Pécs, 33 Landorhegyi Str., H-8900 Zalaegerszeg, Hungary; 4 Institute of Basics of Health Sciences, Midwifery and Health Visiting, Faculty of Health Sciences, University of Pécs, 4 Vörösmarty Str., H-7621 Pécs, Hungary; annamaria.pakai@etk.pte.hu

**Keywords:** Baby-Friendly Hospital Initiative, breastfeeding, exclusive breastfeeding, Hungary

## Abstract

We assessed the prevalence of the “Ten Steps to Successful Breastfeeding” in Hungary and identified possible associations of the steps with breastfeeding. Our quantitative, cross-sectional research was conducted anonymously online in Hungary with a self-administered questionnaire in 2021. Targeted sampling was used, with biological motherhood and having at least one child no older than 60 months as inclusion criteria (*n* = 2008). The implementation of the “Ten Steps to Successful Breastfeeding” was analyzed separately for breastfeeding and non-breastfeeding mothers. A breastfeeding mother was defined as breastfeeding for at least six months. Descriptive statistics, χ^2^ test, and *t*-test were calculated with SPSSv25 (*p* < 0.05). No significant differences were found between breastfeeding and non-breastfeeding mothers in terms of supplementary feeding at the advice of a health professional (*p* = 0.624) and in terms of assistance with breastfeeding or suggested breastfeeding positions during hospitalization (*p* = 0.413). Significant differences were found for receiving breastfeeding-friendly recommendations by staff (*p* = 0.006), valuing breastfeeding (*p* < 0.001), skin-to-skin contact within 1 h (*p* = 0.002), receiving supplementary feeding (*p* < 0.001), rooming-in (*p* < 0.001), responsive feeding, recognizing hunger signs (*p* < 0.001), pacifier/bottle use (*p* < 0.001), and availability of breastfeeding support (*p* = 0.005). Significant differences were observed between breastfeeding and non-breastfeeding subsamples regarding the implementation of baby-friendly steps (*p* < 0.001). Breastfeeding mothers experienced the implementation of more baby-friendly steps and a higher rate of breastfeeding, while there was no significant difference in the duration of exclusive (*p* = 0.795) and partial breastfeeding (*p* = 0.250) based on the results. We concluded that exposure to the Baby-Friendly Hospital Initiative may be associated with increased 6-month breastfeeding but may not influence longer durations.

## 1. Introduction

The Innocenti Declaration was written in 1990 to protect, promote, and support breastfeeding at the “Breastfeeding in the 1990s: Global Initiative” meeting organized by UNICEF/WHO. It emphasizes the uniqueness of breastfeeding and also acknowledges that research has shown its beneficial effects to increase with the duration of breastfeeding [1]. The Declaration states that all mothers should be able to exclusively breastfeed their children for 4–6 months and then continue breastfeeding with appropriate complementary feeding until the child is two years old or beyond. The Declaration highlights the need to strengthen breastfeeding and to resist firmly against a “bottle-feeding culture” in order to achieve this goal: “Efforts should be made to increase women’s confidence in their ability to breastfeed. Such empowerment involves the removal of constraints and influences that manipulate perceptions and behavior towards breastfeeding, often by subtle and indirect means. This requires sensitivity, continued vigilance, and a responsive and comprehensive communications strategy involving all media and addressed to all levels of society. Furthermore, obstacles to breastfeeding within the health system, the workplace and the community must be eliminated” [1]. Measures are necessary to ensure that women receive assistance in maintaining breastfeeding. Additionally, it is suggested that every country’s government develop its own national breastfeeding guidelines and set related goals. These guidelines should be integrated into general healthcare on a national level, and they should support all activities that protect, promote, and support breastfeeding. Regular education for healthcare professionals is necessary to ensure they have the proper knowledge to assist with breastfeeding.

Building on the Innocenti Declaration, the World Health Organisation (WHO) and the United Nations Children’s Fund (UNICEF) jointly recognized the significant impact of postnatal hospital conditions on breastfeeding outcomes and launched the Baby-Friendly Hospital Initiative (BFHI) in 1991.

In the first two steps, measures affecting the management of institutions are set out. Step 1 is closely linked to the WHO International Code of Marketing of Breastmilk Substitutes, which prohibits the promotion and marketing of formula to pregnant women and mothers. Furthermore, they prescribe that maternity institutions must have a written infant feeding regulation, which is routinely communicated to health care workers and parents. In addition, continuous monitoring and data management systems should be established. Step 2 provides for the development of human resources and professional training in order for the staff to have adequate knowledge, competencies, and skills to support breastfeeding. The next eight steps summarize essential clinical practices. Step 3 calls for mothers to be properly informed about the importance and benefits of breastfeeding. In step 4, it is highlighted that the newborn be placed in skin-to-skin contact with its mother immediately after birth and in any case within one hour to allow early breastfeeding. Early breastfeeding within one hour is closely linked to postnatal skin-to-skin contact and avoidance of supplementary feeding [2]. Regarding the fourth step of BFHI, i.e., skin-to-skin contact, one of the most common background factors for early breastfeeding delay is caesarean section [3]. In step 5, the initiation and maintenance of breastfeeding support is advocated as well as the management of common breastfeeding difficulties. Step 6 states not to allow supplementary feeding of the newborn with formula, water, or tea as a substitute for breast milk without a medical indication. The next two steps are closely linked. Step 7 requires that hospital stay should be ensured without restriction through a 24 h rooming-in. Breastfeeding may be negatively affected by the non-rooming-in of the newborn and mother [4]. The implementation of rooming-in, the seventh step of the Baby-Friendly Hospital Initiative, has been positively associated with early skin-to-skin contact and breastfeeding [5]. Step 8 suggests supporting the recognition of newborn hunger signs and promoting responsive feeding. A Hungarian study [6] described the importance of responsive feeding by pointing to the positive correlation between the responsive method and the duration of exclusive breastfeeding.

In step 9, it is recommended to support mothers in breastfeeding and inform them about the risks of using pacifiers and bottles. A study found an association between exclusive breastfeeding cessation, delayed skin-to-skin contact, and pacifier use [7]. At 6 months postpartum, the risk of nonexclusive breastfeeding was lower for infants who were exclusively breastfed for the first 48 h postpartum and were not given a pacifier or bottle [8].

The final, 10th step mandates that institutions coordinate discharge so that parents and their newborns have timely access to ongoing support and care. Once discharged, mothers are best served by a combination of professional and lay support in breastfeeding. Midwives are suitable to inform new mothers about the breastfeeding support available within the community [9].

Overall, the BFHI increases the number and duration of exclusively breastfed infants and is one of the most cost-effective health interventions [10,11,12].

In a study involving 31 maternity hospitals and affiliated polyclinics in Belarus [13], it was found that infants born in Baby-Friendly-Hospital-certified facilities had a significantly higher exclusive breastfeeding rate at 3 months of age (43.3%) than in a control group of hospitals (6.4%) that did not follow the 10 steps to successful breastfeeding. Maternity hospitals are recommended to implement the BFHI [14]. In another study specifically focusing on risk factors of delaying early initiation or not breastfeeding at all, it was found that bottle feeding increased the risk by 18 times, but the lack of rooming-in, complementary feeding, and improper information of mothers about the importance of breastfeeding were also significant determinants [15].

The Innocenti Declaration was supplemented in 2005, emphasizing the global need to develop, enhance, monitor, and evaluate comprehensive infant and young child feeding guidelines, which can be implemented within national programs targeting poor dietary habits, as well as child and reproductive healthcare [16]. It is essential to ensure that the health and related sectors protect, support, and promote exclusive breastfeeding for the first six months and continued breastfeeding for up to two years or beyond while making necessary support available to mothers within the family, community, and workplace. It further emphasizes the promotion of timely, safe complementary feeding alongside continued breastfeeding. Information must be provided regarding infant and young child feeding, especially in challenging circumstances, and the necessary support for mothers, families, and other caregivers must be ensured. Additionally, it advocates for nations to consider what new legal or other measures are needed to enforce the International Code of Marketing of Breast-milk Substitutes and related resolutions by the WHO [17,18,19].

### The Relationship of the Hungarian Recommendation with the BFHI

In addition to supporting and promoting breastfeeding, the BFHI aims to encourage more nations to support their hospitals in achieving the Baby-Friendly label. In Hungary, the first Baby-Friendly Hospital title was given in 2013, and since then, 20 hospitals had held that title; however, the last Baby-Friendly Hospital certification was valid until 2019. Nowadays, the importance of the BFHI lies in the guidelines and recommendations which shapes current maternal and neonatal care in Hungary.

A Hungarian recommendation was summarized in the Ministry of Human Resources (MHR) “Professional Guideline on Feeding Healthy Infants (0–12 months)”, published in 2019, calling for the implementation of the abovementioned 10 steps and compliance with the relevant points of the Code in as many Hungarian maternity facilities as possible. The Hungarian recommendation aims to provide mothers with professional, up-to-date support to help them overcome the difficulties of breastfeeding, thereby increasing the proportion of babies who are exclusively breastfed until approximately six months of age and the proportion of babies who are breastfed with appropriate complementary feeding until one year of age. According to Kopcsó et al., 91.4% of babies born in Hungary between 2018 and 2019 were breastfed by their mothers after birth, and 94.1% of them received breast milk. A total of 76.8% of mothers breastfed their babies responsively [6]. The prevalence of breastfeeding up to six months of age was 53.9%, while the exclusive breastfeeding rate was 25.1%. The Hungarian recommendation [20], based on the BFHI, provides recommendations for the period of hospitalization and discharge, including the need to ensure skin-to-skin contact between mother and newborn for at least one hour after birth, and recommends that routine procedures (bathing and weighing) should be performed only afterward. It mentions the need to ensure the presence of a breastfeeding support professional (nurse/midwife) during the birthing room stay, who can effectively assist the mother–newborn pair in establishing breastfeeding, if necessary, and teach the mother to recognize early signs of hunger and support the success of breastfeeding. It also stresses that substitute fluids (tea, sugar solution, formula, etc.) should only be given for medical reasons. The recommendation also stresses that rooming-in should be ensured. However, it recommends unrestricted, responsive breastfeeding, which should be continued by the mother at home, and avoiding pacifiers to help breastfeeding get off to a good start [21,22,23,24].

Our study’s objective was to assess the prevalence of the “Ten Steps to Successful Breastfeeding” among breastfeeding and non-breastfeeding mothers in Hungary. Furthermore, we aimed to explore the correlations between the number of implemented steps and the rate and duration of breastfeeding.

## 2. Materials and Methods

### 2.1. Data Collection and Sample Characteristics

An observational, quantitative, cross-sectional study was conducted online with a self-administered questionnaire between March and July of 2021. First, a pilot study was conducted with the participation of six mothers to ensure the validity and comprehensibility of the data collecting tool. After finalizing the questionnaire, nonrandom, targeted sampling was used to create the sample. The contact path for the questionnaire was made public on social platforms for mothers. Participation was anonymous and voluntary, with Hungarian mothers being involved. In addition to biological motherhood, the inclusion criteria were at least one child, no older than 60 months, born after the 37th week of pregnancy, and raised in the same household as the mother at the time of the study. We excluded nonbiological mothers, those pregnant with their first child, and those not completing the compulsory questions correctly. We also excluded mothers who reported congenital and/or acquired physical or mental illness of the child or mother that made breastfeeding impractical. A total of 2505 people responded to the questionnaire. After exclusion, 2008 were eligible to participate. From this, we created two subgroups: the breastfeeding group (*n* = 1346) and the non-breastfeeding group (*n* = 662). A mother was considered a breastfeeding participant if she was able to breastfeed the child for at least the first six months of the infant’s life.

### 2.2. Research Design

Our questionnaire and research plan were approved by the Regional/Institutional Scientific and Research Ethics Committee of a University Teaching Hospital in Hungary (nr. 13/2021). Informed consent was obtained from all subjects. Additionally, all methods were carried out in accordance with the Declaration of Helsinki on human subject research.

In the questionnaire, mothers were asked in detail about the mode and events of childbirth as well as the conditions of hospital care and the timing and length of breastfeeding. We divided the participating mothers into two groups for data analysis based on the mode of feeding: a breastfeeding group and a non-breastfeeding group.

We asked the same questions as the “Ten Steps to Successful Breastfeeding” practices outlined in the BFHI (Table 1).

For the baby-friendly steps, there were 10 yes/no and 2 multiple-choice questions to assess the implementation of the BFHI. Most questions were in line with the “Ten Steps to Successful Breastfeeding”. The first two steps were difficult to implement, as the first step relates to whether the maternity care facility has “a written breastfeeding policy that is routinely communicated to all health care staff”. Additionally, the Initiative prohibits the recommendation of infant formula. To assess this, we asked mothers who completed the questionnaire if they had ever given their child formula on the advice of a health professional. The second step is to demonstrate the staff’s knowledge, training, and implementation of baby-friendly practices. To assess this, we asked mothers in the questionnaire whether they were able to have skin-to-skin contact with their baby after birth, whether early breastfeeding (within 1 h) was initiated, and whether the staff recommended responsive feeding. For definition, we used the UNICEF’s description, according to which skin-to-skin contact is when “a baby is dried and laid directly on the mother’s bare chest after birth, both of them covered in a warm blanket and left for at least an hour or until after the first feed” [25]. With these questions, we aimed to assess the staff’s up-to-date knowledge and competencies. The third question is based on the BFHI’s policy to “inform all pregnant women and families about the benefits and management of breastfeeding”. Accordingly, we asked how important they considered breastfeeding their child to be. The fourth step is to “help mothers start breastfeeding within a half-hour of birth”. For this, we asked mothers how long it was after birth that their baby was placed on their chest. In our study, we used the globally used standard for the statistical analysis of this question, i.e., whether early breastfeeding was established within 1 h of birth. For the fifth question, we asked mothers about assistance with proper positioning of the baby at the breast and recommendations regarding different breastfeeding positions. For the sixth step, we collected information on whether the newborn had received supplementary feeding, as the recommendation states that supplementation is only allowed for medical indications. The seventh, eighth, and tenth questions were identical in content, while the sixth and ninth questions were reversed. As for step 7, rooming-in was defined by us as determined by the WHO and UNICEF, as a “hospital practice where postnatal mothers and normal infants stay together in the same room for 24 h a day from the time they arrive in their room after delivery” [16]. For the ninth question, we collected data on the method of feeding to determine whether feeding was carried out with a bottle and to assess pacifier usage habits. Regarding bottle usage, we asked mothers how they fed their baby in the first 6 months, and we requested the appropriate selection from the following response options: exclusive breastfeeding, breast milk from a bottle, breast milk with a feeding aid, breastfeeding with formula supplementation, formula feeding from a bottle, formula feeding with a feeding aid, breastfeeding with introduction of complementary foods, formula feeding with introduction of complementary foods, or breastfeeding with formula feeding and introduction of complementary foods. Additionally, we asked mothers whether they offered a pacifier or comforter to their baby. In the tenth step, we asked whether they received information upon discharge from the hospital about whom to contact if they encountered breastfeeding difficulties at home.

### 2.3. Statistical Analysis

IBM SPSS 25 was used for statistical analysis. Descriptive statistics were used to analyze the distribution of the steps. The association of breastfeeding with several factors and baby-friendly steps was examined using the χ^2^ test. Differences in the duration of breastfeeding and exclusive breastfeeding between the two groups created were examined using independent samples *t*-tests and Mann–Whitney U tests. The significance level was set at *p* < 0.05 [26].

## 3. Results

The characteristics of breastfeeding and non-breastfeeding mothers, according to different sociodemographic factors, can be seen in Table 2.

No significant differences were found between the breastfeeding and non-breastfeeding mothers in terms of supplementary feeding at the advice of a health professional (*p* = 0.624).

There was a significant difference among the subgroups in terms of the health professionals’ recommendations advocating breastfeeding, with nearly twice as many people in the breastfeeding group reporting that the health worker recommended skin-to-skin contact or responsive feeding than in the non-breastfeeding group (*p* = 0.006). However, within the breastfeeding group, a 10% higher proportion of those who were breastfed were breastfed if a health professional had suggested supporting steps. There was a significant difference between the breastfeeding and non-breastfeeding groups in terms of the importance of breastfeeding, with those who felt it important to breastfeed doing so almost 40% more often (*p* < 0.001).

Skin-to-skin contact was significantly higher in the breastfeeding group. Two-thirds of mothers who breastfed had skin-to-skin contact with their baby after birth compared to less than one-third of mothers who did not breastfeed (*p* = 0.002). In addition, mothers who had skin-to-skin contact with their baby within the first 1 h of birth had a 10% higher rate of breastfeeding.

However, no association was detected between assistance with breast positioning or recommended breastfeeding positions during hospitalization for the two groups (*p* = 0.413). Within the breastfeeding group, those who received assistance with correct breast positioning and learning different breastfeeding positions during hospitalization had a higher breastfeeding rate of only 2.2%.

We found a significant difference in the rate of supplementary feeding of newborns in the breastfed and non-breastfed groups (*p* < 0.001). There was a significantly higher rate of non-supplementary feeding of newborns in the breastfed group than in the non-breastfed group. Furthermore, a 20% higher proportion of infants who were not supplementally fed were breastfed.

There is a significant difference between the two groups in terms of rooming-in. It was significantly higher in the breastfed group (almost 70%) and in the non-breastfed group (just over 30%) (*p* < 0.001). Furthermore, the proportion of breastfeeding mothers in the rooming-in group was 15% higher.

A significantly higher proportion of the breastfed group reported responsive feeding and/or being taught in the hospital how to recognize hunger signs in their child (*p* < 0.001). Nearly 30% higher rates of breastfeeding were found in responsive feeding and/or mothers taught to recognize hunger signs. Nearly three-quarters of mothers who were instructed in responsive feeding or in recognizing their child’s hunger cues breastfed their child, while this ratio was less than half of mothers from the other group.

Additionally, the proportion of children in the breastfed group who did not use a pacifier/bottle was significantly higher (*p* < 0.001). However, children who did not use either a bottle or a pacifier were 10% more likely to be breastfed than those who used either device.

In the breastfeeding group, significantly more mothers said that they were contacted upon discharge from the hospital if they had difficulty breastfeeding (*p* = 0.005). In addition, those who were contacted for breastfeeding support were 6.5% more likely to breastfeed.

There was a significant difference between the breastfeeding group and the non-breastfeeding group in terms of the number of baby-friendly steps taken (*p* < 0.001) (Table 3).

**Figure 1:**
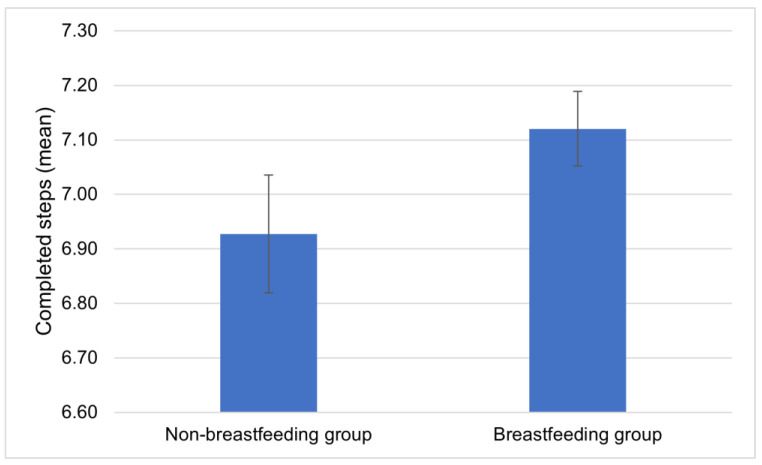
Average number steps completed by the breastfeeding (*n* = 662) and non-breastfeeding (*n* = 1346) mothers.

In total, 69.5% (*n* = 889) of the breastfeeding group compared to only 30.5% (*n* = 391) of the non-breastfeeding group reported having taken seven or more baby-friendly steps during their hospital stay. The non-breastfeeding group achieved an average of 6.92 (SD = 1.35; 95% CI: 6.82–7.04; min. 2, max. 10), while the breastfed group achieved 7.12 (SD = 1.21; 95% CI: 7.05–7.19; min. 1, max. 10) baby-friendly steps (Figure 1). In addition, more than seven practices are positively associated with a 9% higher rate of breastfeeding, as 60.5% (*n* = 308) of those who experienced less than that were breastfeeding.

In addition, mothers in the breastfeeding group who accomplished at least seven baby-friendly steps during hospitalization did not differ significantly from mothers who experienced fewer than seven baby-friendly steps in terms of the duration of exclusive breastfeeding, which lasted on average for almost 5 months (*p* = 0.250), or the duration of breastfeeding, which lasted on average for more than 10 months (*p* = 0.795) for both groups (Table 4).

We also examined whether the implementation of baby-friendly steps available in the hospital influenced women’s expectations and practices regarding breastfeeding. We compared the responses to the question “Did you breastfeed for as long as you originally planned?” with the answers indicating the implementation of baby-friendly steps and obtained the results as shown in Table 5.

## 4. Discussion

A significant proportion of participants reported taking steps to implement the Baby-Friendly Hospital Initiative at the time of birth. The Innocenti Declaration (1990) advocates exclusive breastfeeding for 4–6 months and breastfeeding until at least two years of age, with appropriate complementary feeding. Based on the mothers’ reports, our study found that the recommendations for exclusive breastfeeding were met, but the average duration of breastfeeding was 10 months, which was below the recommendation. The “Ten Steps to Successful Breastfeeding” were achieved at a higher rate among breastfeeding mothers (*p* < 0.001). Step 1 of the BFHI prohibits the advertising of infant formula in maternity facilities, so we asked mothers in our questionnaire if they had ever fed their babies formula on the advice of a health professional. Our survey found no association regarding the recommendation of formula in either group (*p* = 0.624). To assess the practical implementation of BFHI step 2, which reflects the up-to-date knowledge of health professionals in clinical practice regarding the steps, mothers were asked whether they were allowed to remain in skin-to-skin contact with their baby after birth or whether staff suggested responsive feeding. The results show that more than twice as many mothers in the breastfeeding group reported that at least one of these conditions was met at birth. However, even within the breastfeeding group, the difference was significant, with a nearly 10% higher proportion of those who breastfed meeting at least one of these conditions (*p* = 0.006). The aim of step 3 is to increase mothers’ and their families’ knowledge about the importance of breastfeeding and thereby encourage them to breastfeed. In our study, more than two-thirds of breastfeeding mothers considered it important, while the majority of the non-breastfeeding subsample, also two-thirds, did not consider it important to be able to breastfeed their child (*p* < 0.001). Step 4 (skin-to-skin contact within 1 h and breastfeeding) was significantly higher in the breastfeeding group (*p* = 0.002). Our results thus support the claim of Ahmed et al. that early skin-to-skin contact and early breastfeeding are positively related [2].

Step 5 includes professional breastfeeding support in hospital care, which was assessed by asking mothers whether they were helped to breastfeed correctly and whether they were advised of different breastfeeding positions. Our results show that although a higher proportion of the breastfeeding group received assistance with breastfeeding and/or were suggested different breastfeeding positions, the difference was not significant (*p* = 0.413). In addition, among the breastfeeding group, only 2.2% less who received assistance and/or were referred to different breastfeeding positions chose to breastfeed. Step 6, the avoidance of supplementary feeding, was met at a significantly higher rate in the breastfeeding group. The highest proportion, three-quarters of breastfeeding mothers, reported that their child did not receive supplementary feeding, while the highest proportion of non-breastfeeding mothers reported that their child received supplementary feeding after birth (*p* < 0.001). Step 7 highlights rooming-in placement, which was significantly higher in the breastfeeding group (*p* < 0.001). A higher rate of non-rooming-in placement was observed among non-breastfeeding mothers. Thus, the conclusion of Cadwell et al. that breastfeeding may be negatively affected by non-rooming-in placement and the finding of Hakala et al. that rooming-in and breastfeeding are associated were supported by our research findings [4,5]. In addition to the importance of rooming-in, the BFHI also highlights the importance of responsive breastfeeding in step 8. Several well-founded scientific studies have aimed to provide evidence regarding whether rooming-in placement affects indicators measuring the success of breastfeeding, even in the long term (>3 months). However, high-quality results regarding this are still limited [27,28]. We hope that our research will contribute to increasing the reliability and validity of results in this regard.

In Hungary, the professional guidelines of the Ministry of Human Resources [20] also recommend responsive breastfeeding. In our survey, we asked mothers about the implementation of responsive feeding and how they recognize their child’s hunger signals. Three-quarters of mothers in the breastfeeding group reported responsive feeding or being able to recognize their child’s hunger cues, while only one-quarter of mothers in the non-breastfeeding group reported the same (*p* < 0.001).

Step 9 of the BFHI suggests promoting breastfeeding among mothers and informing them about the risks of bottle and pacifier utilization. The Hungarian MHR guideline [20] does not recommend pacifiers and bottles for infants. Significantly more of the breastfeeding group reported that their infant did not use a pacifier or bottle for feeding, while only one-quarter of the non-breastmilk-feeding group reported the same (*p* < 0.001). The last step, giving contact information during hospital discharge in case of breastfeeding difficulties, was implemented at a significantly higher rate in the breastfeeding group. Our results thus support Thorley’s findings that mothers discharged are best supported in breastfeeding by the support and information they receive from midwives [9]. Achieving seven or more baby-friendly steps was found to be associated with a higher proportion of breastfeeding mothers in the hospital than non-breastfeeding mothers (*p* < 0.001). Mothers who used breastmilk to feed their children experienced the implementation of more baby-friendly measures and a higher rate of breastfeeding occurrence in their cases. However, there was no significant difference in the length of exclusive (*p* = 0.795) and partial breastfeeding (*p* = 0.250).

A limitation of the research is that the population of breastfeeding and non-breastfeeding mothers seemed to be significantly different in some socio-demographic factors, e.g., marital status or educational attainment.

The protocol for complementary feeding varies between maternity units, and in some cases, it is not necessarily used. Additionally, the sample surveyed in our study is by no means representative. The fact that the survey was limited to mothers who have access to the Internet and are able to use it may be a source of bias. Also, undetected errors and recall biases may occur due to the fact that all of our data are self-reported. In Hungary, there is no available database about information on obstetric interventions, postpartum care for the mother and newborn, and their environment. Therefore, we can only gather self-reported data, which is why we opted for online data collection to achieve a higher number of responses. Furthermore, as the WHO recommends breastfeeding exclusively for the first 6 months, followed by appropriate complementary feeding up to 2 years or beyond, we tried to determine the inclusion criteria to include those who breastfeed their children for more than 2 years while minimizing recall bias. Thus, we established the age of 60 months as the cutoff, ensuring that the child was still preschool-aged.

## 5. Conclusions

Our research results suggest that exposure to the Baby-Friendly Hospital Initiative’s “Ten Steps to Successful Breastfeeding” may be associated with a higher incidence of 6-month breastfeeding, but may not influence longer durations.

We recommend establishing a national-level database in Hungary and those countries where it does not exist, which would enable us to track interventions and postpartum events related to the same birth. Conducting routine quantitative data collection on a representative sample would allow for the analysis of clinical parameters to assess the short- and long-term effects of obstetric interventions and postpartum care conditions.

There is a significant role in expanding the training and up-to-date knowledge of healthcare professionals in contributing to successful breastfeeding. Therefore, we propose the creation of a series of interactive and informative programs compiled through the joint organization of midwives, health visitors, dietitians, as well as mental health professionals. These presentations would support the preparation for parenthood, childbirth, and breastfeeding based on the joint professionals’ individual competencies and practical experiences. This way, (expectant) mothers would gain knowledge from professionals about international and national guidelines, including the Baby-Friendly Hospital Initiative, thus expanding their understanding of the significance and benefits of breastfeeding as well as risk factors adversely affecting it.

## Figures and Tables

**Table 1:** Comparison of the BFHI’s ten steps and the questions used during the survey.

Ten Steps to Successful Breastfeeding [16]	Questions Used during the Survey
Step 1: Have a written breastfeeding policy that is routinely communicated to all health care staff.	Have you ever given infant formula, recommended by a health care staff?
Step 2: Ensure that staff have sufficient knowledge, competence and skills to support breastfeeding.	Was responsive breastfeeding recommended to you?
Step 3: Discuss the importance and management of breastfeeding with pregnant women and their families.	Was it important to you to be able to breastfeed?
Step 4: Facilitate immediate and uninterrupted skin-to-skin contact and support mothers to initiate breastfeeding as soon as possible after birth.	After birth, how much time passed until your child was placed on your chest?
Step 5: Support mothers to initiate and maintain breastfeeding and manage common difficulties.	Were you helped with breast positioning in the hospital? Were different breastfeeding positions recommended to you in the hospital?
Step 6: Do not provide breastfed newborns any food or fluids other than breast milk, unless medically indicated.	Did your child receive supplementary feeding with or instead of breast milk after birth?
Step 7: Enable mothers and their infants to remain together and to practise rooming-in 24 h a day.	Mark the occurrence of the following conditions: rooming-in (I was co-placed with my baby in the hospital).
Step 8: Support mothers to recognize and respond to their infants’ cues for feeding.	In the hospital, was it recommended to breastfeed responsively, and were you informed about how to recognize your child’s hunger cues?
Step 9: Counsel mothers on the use and risks of feeding bottles, teats, and pacifiers.	How do/did you feed your child in the first 6 months? Has your child ever received a pacifier?
Step 10: Coordinate discharge so that parents and their infants have timely access to ongoing support and care.	In the hospital, did they give you information about who you can contact if you have difficulties breastfeeding at home?

**Table 2:** Proportion of breastfeeding (*n* = 1346) and non-breastfeeding (*n* = 662) mothers in different sociodemographic factors.

		Non-Breastfeeding Group (%)	Breastfeeding Group (%)	*p*
Mother’s age at birth	<25	17%	11%	
	25–29	34%	35%	
	30–34	32%	36%	<0.001
	>34	17%	19%	
Residence	Village	32%	27%	
	City	55%	51%	<0.001
	Capital	13%	22%	
Marital status	Single	4%	5%	
	Registered partnership	13%	6%	
	Married	81%	87%	<0.001
	Divorced	2%	1%	
Educational attainment	Primary school or less	2%	1%	
	High school	43%	27%	
	Vocational school	8%	3%	<0.001
	College/University	47%	69%	
Average net income per capita per month (HUF)	40.000–160.000	45%	33%	
	161.000–330.000	40%	50%	<0.001
	>330.000	15%	17%	
Parity	1	71%	64%	
	2	22%	28%	0.014
	≥3	7%	8%	
The answers are based on which children?	First	77%	72%	
	Second or more	23%	28%	0.023
Child’s sex	Boy	53%	50%	
	Girl	47%	50%	0.296
Mode of birth	Natural	56%	65%	
	Planned caesarean section	17%	12%	0.195
	Emergency caesarean section	27%	24%	

**Table 3:** Comparison of the breastfeeding and non-breastfeeding groups according to the ten steps.

Questions Measuring BFHI’s Steps		Non-Breastfeeding Mothers *n* (%)		Breastfeeding Mothers *n* (%)	Total (%)	χ^2^ (1)	*p*
Step 1	Used formula recommended by staff	No	630 (33.1%)	1274 (66.9%)	1904 (100%)	0.24	0.624
		Yes	32 (30.8%)	72 (69.2%)	104 (100%)		
Step 2	Had skin-to-skin contact or were advised by staff to breastfeed responsively	No	97 (41.6%)	136 (58.4%)	233 (100%)	7.572	0.006
		Yes to either	551 (32.5%)	1142 (67.5%)	1693 (100%)		
Step 3	Considered breastfeeding important	No	98 (68.1%)	46 (31.9%)	144 (100%)	86.419	<0.001
		Yes	564 (30.3%)	1300 (69.7%)	1864 (100%)		
Step 4	Had skin-to-skin contact and breastfed her child within one hour of birth	No	145 (39.4%)	223 (60.6%)	368 (100%)	9.74	0.002
		Yes to either	499 (31%)	1113 (69%)	1612 (100%)		
Step 5	Was helped in proper positioning OR was advised to use different breastfeeding positions in the hospital	No	139 (35.4%)	254 (64.6%)	393 (100%)	0.671	0.413
		Yes to either	514 (33.2%)	1035 (66.8%)	1549 (100%)		
Step 6	The newborn received supplementary feeding beside or instead of breast milk after birth	No	240 (23.3%)	791 (76.7%)	1031 (100%)	90.703	<0.001
		Yes	383 (43.8%)	491 (56.2%)	874 (100%)		
Step 7	Got rooming-in placement in the hospital	No	65 (47.4%)	72 (52.6%)	137 (100%)	14.206	<0.001
		Yes	591 (31.8%)	1269 (68.2%)	1860 (100%)		
Step 8	Breastfed responsively OR was informed about hunger cues	No	58 (53.2%)	51 (46.8%)	109 (100%)	39.268	<0.001
		Yes to either	400 (25.5%)	1166 (74.5%)	1566 (100%)		
Step 9	The child received bottle OR pacifier	No	41 (25%)	123 (75%)	164 (100%)	6.131	0.013
		Yes to either	612 (34.6%)	1159 (65.4%)	1771 (100%)		
Step 10	Was given contact in case of breastfeeding difficulties	No	473 (35.7%)	852 (64.3%)	1325 (100%)	7.903	0.005
		Yes	180 (29.2%)	436 (70.8%)	616 (100%)		

**Table 4:** Duration of (exclusive) breastfeeding regarding the number of accomplished steps.

Steps	<7		≥7		*t* (df)	*p*
	M	SD	M	SD		
Duration of exclusive breastfeeding (months)	4.91	2.33	4.94	2.26	−0.259 (1475)	0.795
Duration of breastfeeding (months)	10.19	8.73	10.70	7.96	−1.15 (1730)	0.250

**Table 5:** Differences in the intended breastfeeding duration according to the steps achieved.

Steps Reported as Achieved		“Did You Breastfeed Your Child for as Long as You Originally Planned?”		
	Yes (*n* (%))	No (*n* (%))	χ^2^ (1)/Z	*p*
Step 1	1237 (74%)	426 (26%)	11.631	0.009
Step 2	884 (76%)	281 (24%)	3.689	0.055
Step 3	1291 (77%)	391 (23%)	52.068	<0.001
Step 4	105 (63%)	63 (38%)	−2.757	0.006
Step 5	960 (74%)	332 (26%)	0.033	0.857
Step 6	804 (82%)	171 (18%)	59.904	<0.001
Step 7	1259 (76%)	388 (24%)	21.723	<0.001
Step 8	525 (74%)	189 (26%)	0.648	0.421
Step 9	763 (88%)	102 (12%)	154.161	<0.001
Step 10	422 (76%)	136 (24%)	0.544	0.461

## Data Availability

The data presented in this study are available on request from the corresponding author. The data are not publicly available due to privacy restrictions.
